# The feasibility of assessing frailty and sarcopenia in hospitalised older people: a comparison of commonly used tools

**DOI:** 10.1186/s12877-019-1053-y

**Published:** 2019-02-15

**Authors:** Kinda Ibrahim, Fiona F. A. Howson, David J. Culliford, Avan A. Sayer, Helen C. Roberts

**Affiliations:** 1 Academic Geriatric Medicine, University of Southampton, Southampton, UK; 2 National Institute for Health Research Collaboration for Leadership in Applied Health Research and Care (NIHR CLAHRC) Wessex, University of Southampton, Southampton, UK; 3 University Hospital Southampton NHS Foundation Trust, Southampton, UK; 4 AGE Research Group, Institute of Neuroscience, Newcastle University, Newcastle, UK; 5 NIHR Newcastle Biomedical Research Centre, Newcastle upon Tyne Hospitals NHS Foundation Trust and Newcastle University, Newcastle, UK

**Keywords:** Older, Hospital, Frail, Sarcopenia, Assessment, Feasibility, Grip strength, Muscle mass, Gait speed

## Abstract

**Background:**

Frailty and sarcopenia are common amongst hospitalised older people and associated with poor healthcare outcomes. Widely recognised tools for their identification are the Fried Frailty Phenotype, its self-report version the FRAIL Scale, and the European Working Group on Sarcopenia in Older People (EWGSOP) criteria. We studied the feasibility of using these tools in a hospital setting of acute wards for older people.

**Methods:**

Patients aged 70+ years admitted to acute wards at one English hospital were prospectively recruited. The Fried Frailty Phenotype was assessed through measured grip strength, gait speed and questions on unintentional weight loss, exhaustion and physical activity. The 5-item self-reported FRAIL scale questionnaire covering the same domains was completed. Agreement between the two tools was reported using the Cohen kappa statistic. The EWGSOP criteria (gait speed, grip strength and muscle mass) were assessed by additional bedside measurement of muscle mass with bioelectrical impedance.

**Results:**

Two hundred thirty three participants (median age 80 years, 60% men) were recruited. Most (221, 95%) had their grip strength measured: 4 (2%) were unable and data were missing for 8 (3%). Only 70 (30%) completed the gait speed assessment: 153 (66%) were unable with missing data on 10 (4%). 113 (49%) participants had the bioelectrical impedance assessment. Muscle mass measurement was not possible for 84 (36%) participants: 25 patients declined, 21 patients were unavailable, 22 results were technically invalid, and 16 had clinical contra-indications. Data on 36 (15%) were missing.

Considering inability to complete grip strength or gait speed assessments as low values, data for the Fried Frailty Phenotype was available for 218 (94%) of participants; frailty was identified in 105 (48%). 230 (99%) patients completed the FRAIL scale; frailty was identified among 77 (34%). There was moderate agreement between the two frailty tools (Kappa value of 0.46, 95%CI: 0.34 to 0.58). Complete data for the EWGOSP criteria were only available for 124 (53%) patients of whom 40 (32%) had sarcopenia.

**Conclusion:**

It was feasible to measure grip strength and complete the FRAIL scale among older inpatients in hospital. Measuring gait speed and muscle mass to identify sarcopenia was challenging in the acute setting.

**Trial registration:**

ISRCTN registry (ID ISRCTN16391145) on 30.12.14.

## Background

Frailty (multi-system impairment associated with increased vulnerability to stressors) and sarcopenia (muscle loss, weakness and reduced muscle function associated with ageing) are both associated with adverse health outcomes and admission to hospital [1, 2]. Sarcopenia has been recently recognized in the International Classification of Diseases (ICD-10) as a condition that should be diagnosed in older populations [3]. There is also increasing recognition of the importance of identifying frailty, but identifying these common conditions is neither routine nor standardised in the in-patient hospital setting [4, 5]. This is an important area to address in view of the prevalence, reversibility, and prognostic value of these two conditions [6].

There are many reported objective and subjective frailty measurement methods with the Fried Frailty Phenotype a widely used tool to identify frailty and reported in 70% of published papers [7]. A recent survey of 388 clinicians from 44 countries reported that the Fried Frailty Phenotype (27%), gait speed assessment (44%), and the Clinical Frailty Scale (34%) are among the most widely used frailty tools [8]. The five item self-report frailty tool “FRAIL scale” developed from the Fried Frailty Phenotype does not require physical measurements of grip strength or gait speed [9] and is recommended by the International Academy on Nutrition and Aging for use in daily clinical practice [10, 11].

The European Working Group in Sarcopenia in Older People (EWGSOP) algorithm based on physical measurements of gait speed, grip strength and muscle mass is widely recognised for the diagnosis of sarcopenia in clinical settings including hospitals [12]. Validated questionnaire-based screening methods for sarcopenia such as the SARC-F questionnaire, are more suitable for screening to detect those at-risk of sarcopenia in primary care settings [13, 14].

It has been suggested that most of the definitions and tools to measure frailty and sarcopenia are more suited to research studies than wider clinical practice [15]. Recent work in the UK reported that it was feasible and acceptable to routinely measure grip strength of older people (70+ years) in hospital [16]. Jadczak et al. reported that use of the FRAIL scale was feasible in hospitalised frail older adults [17]. However, no study has yet investigated the feasibility of assessing both syndromes together in clinical practice. The aim of this study was to assess whether it is feasible to assess both frailty and sarcopenia, with a focus on the physical measurements central to the Fried Frailty Phenotype and EWGSOP criteria, among older people in hospital.

## Methods

### Study design and setting

This cross-sectional prospective study was part of a larger study of mealtime volunteers [18].

### Participants

Patients aged 70 years and over admitted as acute admissions to one large English hospital were eligible. Patients were recruited prospectively over a 2-year period from March 2014 to March 2016. Patients were excluded if they were receiving artificial nutrition (enteral or parenteral), nursed in a side room, on an end of life pathway or unable to consent. All participants gave informed, written consent.

### Data collection

Demographic data including date of birth, gender, marital status and usual living arrangements were abstracted from participants’ clinical records. Active co-morbidities were obtained from medical records and used to calculate the Charlson Comorbidity Index. Participants’ regular prescribed medication was determined from the computerised prescribing system on the day of their inclusion into the study. Cognition and mood were assessed using the Mini Mental State Examination (MMSE) (a score of < 24/30 indicates cognitive impairment) [19] and the 15 item Geriatric Depression Scale (GDS) (a score > 5 indicates that depression is likely) [20]. The most recent Body Mass Index (BMI) was abstracted from the clinical records. Physical activity was measured using the Physical Activity Scale for the Elderly (PASE) questionnaire which assesses the duration, frequency, exertion level, and amount of physical activity undertaken over a 7 day period by individuals 65 years and older [21]. Activities of Daily Living (ADL) were measured using the 11 –item Modified Barthel Index [22]. Length of hospital stay, discharge destination, readmissions and death after 6 months were abstracted from clinical records.

#### Assessment of frailty

Frailty was measured using the Fried Frailty Phenotype and the FRAIL scale.

The Fried Frailty Phenotype identifies frailty by the presence of three or more of self-reported weight loss of >10lbs (4.5 kg) in the last year, exhaustion, low physical activity, slow gait speed and weak grip strength [23]. Exhaustion was determined by self-report of feeling that “in the last week, everything was an effort or I could not get going” for three or more days. Physical activity level was considered low if the participant’s PASE score was in the lowest quintile, based on age and gender. Maximum grip strength was measured using a Jamar dynamometer and standardised protocol [24]. Low grip strength was defined as < 20 kg (kg) for women and < 30 kg for men [12]. Normal gait was timed over a 4 m course and low gait speed defined as < 0.8 m/second (m/s) [12]. Usual practice in published studies of reporting an inability to complete grip strength or gait speed measurement as low values was followed [25, 26].

The FRAIL scale includes 5 questions assessing fatigue, resistance, ambulation, illness and loss of weight [27]. Each question scores one point with frailty identified if participants score 3–5 points; 1–2 points represents pre-frailty and 0 points a robust health status similar to the Fried Frailty Phenotype.

#### Assessment of sarcopenia

The EWGSOP criteria of normal gait speed and grip strength were previously measured as outlined above. Muscle mass was measured using a portable bioelectrical impedance machine (ImpediMedSFB7), a simple, non-invasive technique. Skeletal muscle mass was calculated using Janssen’s formula and considered to be low when the skeletal muscle index was below the cut-off values validated in older people: SMI < 8.87 kg/m2 and < 6.67 kg/m2 in men and women, respectively [28].

### Data analysis

The focus of this study was on the physical measurements of frailty and sarcopenia criteria including grip strength, gait speed, and muscle mass. The primary outcome was the proportion of patients who a) had performed each measurement, b) were unable to complete them or c) had missing data. The analysis was mainly descriptive. Counts and percentages were reported for categorical variables. The median and inter-quartile range were used to summarise all continuous variables, none of which were normally distributed. The Kappa statistic was used to assess agreement between the Fried Frailty Phenotype and FRAIL Scale, for which 95% confidence intervals (95% CI) were estimated. Tests of statistical significance were not carried out due to the descriptive nature of the analysis. All statistical analyses were performed in Microsoft Excel 2003 (Microsoft Corporation, Redmond, WA) and SPSS version 24 (SPSS software, IBM Corporation, Armonk, NY).

## Results

### Participants’ characteristics

Two hundred thirty three participants (median age 80 years, 60% men) were recruited to the study (Table 1). Most (94%) participants lived in their own home and half were married. The median number of comorbidities and the median Charlson Comorbidity Index score was 5, with a median of 8 medications per patient. Median cognition, mood and physical function (ADL) were within normal values. The median length of stay was 10 days, and most participants (77%) were discharged to their usual residence. Only 3% of participants died during the following 6 months.

**Table 1:** Participants’ demographic and clinical characteristics

	Participants (*n* = 233)
Age	80 (75,86)
Gender, n (%)	
Male	139 (60)
Female	94 (40)
Marital status, n (%)	
Single or Divorced/Separated	33 (14)
Married or Cohabiting	114 (49)
Widowed	86 (37)
Usual residence, n (%)	
Private home	220 (94)
Warden-supported housing	8 (3)
Nursing / Care home	5 (4)
Number of comorbidities (*n* = 196, 84.1%)	5 (3,7)
Charlson Comorbidity Index	5 (4,7)
Number of medications (*n* = 196, 84.1%)	8 (6,11)
BMI (*n* = 232, 99.6%)	25.8 (22.6,28.6)
MMSE (*n* = 232, 99.6%)	28 (25,29)
GDS (*n* = 231, 99.1%)	3 (2,5)
PASE	51 (25, 107.5)
Barthel	87 (70,100)
Length of stay (*n* = 226, 97.0%)	10 (4,19)
Discharge destination, n (%)	
Usual residence	174 (77)
New care home	19 (8)
Rehabilitation	19 (8)
Another hospital	8 (4)

*n* number of participants (stated for variables where less than complete data was observed), *%* percentage; Summary statistics are median and inter-quartile range, unless otherwise stated. *BMI* Body Mass Index, *MMSE* Mini Mental State Examination, *GDS* Geriatric Depression Scale, *PASE* Physical Activity Scale for the Elderly

### Feasibility of frailty and sarcopenia assessment

Two hundred twenty one of the 233 participants (95%) completed the grip strength measurement and 157 (67%) had low grip strength (Table 2). Only 4 (2%) participants were unable to complete the measurement and data were missing for 8 (3%). However, the majority of participants (153, 66%) were unable to undertake the gait speed assessment with missing data for a further 10 (4%). Among the 70 (30%) who were able to walk four metres, 27 had a slow gait speed and 43 had normal gait values. 113 (49%) participants completed the bioelectrical impedance assessment, of whom 66 had low values. 84 (36%) of participants did not complete the muscle mass assessment test for different reasons including: patient declined (25, 30%), technical difficulties with the machine (22, 26%), clinical contraindication (16, 19%), and patient unavailable, for example engaged in clinical activities (21, 25%). Missing data for the bioelectrical impedance assessment was recorded in 15%.

**Table 2:** Completion of physical assessments

	Normal	Low	Missing /no reason given	Unable /declined
Grip strength	64 (28%)	157 (67%)	8 (3%)^a^	4 (2%)^a^
Gait speed	43 (18%)	27 (12%)	10 (4%)^a^	153 (66%)^a^
Muscle mass	47 (21%)	66 (28%)	36 (15%)^a^	84 (36%)^ab^

^a^Subjects for whom the assessment was not completed (missing/no reason given/unable/declined) comprised: grip strength - 6 female, 6 male; gait speed measurements - 77 female, 86 male; and muscle mass measurements - 47 female, 73 male

^b^Patients declined *n* = 25, technical difficulty with machine *n* = 22, patient unavailable *n* = 21, contraindication (pacemaker, leg ulcers, etc.) *n* = 16

The Fried Frailty Phenotype was assessed in 218/233 (94%), considering the inability to complete the grip strength or the gait speed measurement as a low value. 230/233 (99%) participants were able to complete the self-reported Frail scale questionnaire. Data for sarcopenia assessment were available for 124/233 (53%) patients only, reflecting the difficulty with measuring muscle mass in this group.

### Prevalence of frailty and sarcopenia

The Fried Frailty Phenotype identified 105/218 (48%) participants as frail, (Table 3) while the FRAIL scale identified only 77/230 (34%) patients as frail. 46% of patients were found to be pre-frail using both tools. However, 6 and 20% of patients were found robust using the Fried Frailty Phenotype and FRAIL scale, respectively. There was moderate agreement between the Fried Frailty Phenotype and FRAIL scale (Kappa value of 0.46, 95%CI: 0.34 to 0.58).

**Table 3:** Prevalence of frailty and sarcopenia

	n (%)
Fried Frailty Score (*n* = 218)	
Not frail (0)	12 (6)
Pre-frail (1 to 2)	101 (46)
Frail (3 and over)	105 (48)
FRAIL scale (*n* = 230)	
Not frail (0)	47 (20)
Pre-frail (1 to 2)	106 (46)
Frail (3 and over)	77 (34)
Sarcopenia EWGOSP (*n* = 124)	
NO	84 (68)
Yes	40 (32)

The EWGSOP criteria identified 40/124 (32%) patients with sarcopenia among those who completed the bioelectrical impedance muscle mass assessment. There was only slight agreement between Fried Frailty Phenotype and sarcopenia (Kappa of 0.14, 95% CI: 0 to 0.33).

**Fig. 1:**
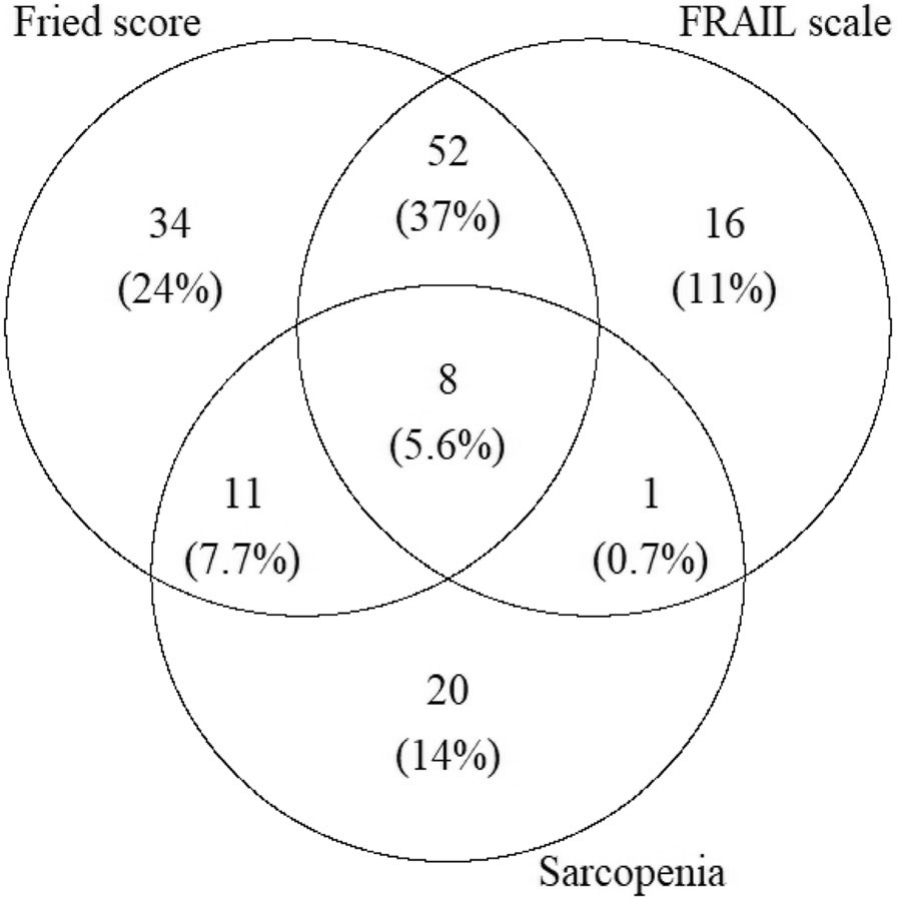
A Venn diagram showing the overlap between the two different frailty criteria and sarcopenia

60 (43%) patients were found to have frailty using both the Fried frailty phenotype and FRAIL scale, and 20 (14%) patients had both frailty (using either tool) and sarcopenia see Fig. 1.

## Discussion

The aim of this study was to assess whether it is feasible to identify frailty and sarcopenia amongst older people in hospital using the measurements required to underpin the widely validated Fried Frailty Phenotype and EWGSOP criteria. It was feasible to measure grip strength in most (95%) participants, but we could measure gait speed in only 30% and muscle mass in only 50% of participants even using bedside techniques. Research evidence supports considering inability to complete grip strength and gait speed assessment as low values, and so frailty could be identified using the Fried Frailty Phenotype. However such evidence is unavailable for muscle mass measurement, so sarcopenia was challenging to assess in this hospital population using the EWGSOP criteria. The prevalence of frailty in this group was 34% (using the FRAIL Scale completed by 99% participants) and 48% (by the Fried Frailty Phenotype completed by 92% participants). The prevalence of sarcopenia was 32% among those for whom data were available (53% participants).

High levels of completion of grip strength measurement have also been reported in a Danish study of 52 acute medical inpatients (mean age 78 years) [29] and among 811 UK acute medical patients aged 70+ years [15]. Confusion, dementia, patient refusal, severe acute illness, aggressive patients, and severe arthritis were among the reported reasons for inability to measure grip strength [16]. Low grip strength was found in 71% of patients in our study which is similar to that reported by other studies [16, 30].

Gait speed is potentially easy to measure and is not limited to a specific health care discipline [15, 31]. However, measuring gait speed among older inpatients in our study was challenging and only 30% of our participants were able to walk four metres. North American studies of 35 patients aged 65+ in the Emergency Department and 322 older medical inpatients (mean age 76 years) reported 60 and 64% completion rates respectively [32, 33]. An Italian study of 103 hospital inpatients (mean age 84 years) reported that 22.3% were unable to perform either gait speed or handgrip measurements [34].

A recent international survey of 255 clinicians from 55 countries reported that 58% of clinicians that measure muscle mass in their practice use anthropometric data (body mass index, calf circumference, mid-upper arm circumference and skinfold thickness) and 46% use dual-energy X-ray absorptiometry (DXA) [35]. However, anthropometric measures are prone to error and less suitable for assessing muscle mass in older people [36] while inpatients may find it difficult to undergo DXA [37] which also has associated costs and requirement for trained assessors and equipment limiting routine use [38]. Bioelectrical impedance analysis is suggested for muscle mass assessment in primary care setting [39] but in this study bioelectrical impedance was only feasible among half of older inpatients. It can be contraindicated (examples include leg ulcers, pacemaker), challenging due to patient factors (unavailability or declining) or for technical reasons (rejected or invalid readings), and is sensitive to subjects’ hydration and recent activity [40]. In our study patient factors (decline, unavailable) and clinical/technical factors were fairly equally responsible for lack of muscle mass measurement. Other studies of sarcopenia in hospital inpatients reported higher completion with around 58–90% participants able to have bioelectrical impedance assessment [41, 42]. These studies recruited included elective admissions and younger patients and had a shorter median length of stay, which could imply those participants were more able to comply with the assessment. Technical problems could be minimised by regular calibration of the machines. Patient factors could be addressed by increasing public awareness of sarcopenia. The guidelines for the diagnosis of sarcopenia (EWGSOP2) have recently been updated. They now recommend case-finding using a brief questionnaire such as the SARC-F [43], and then focus on low muscle strength as the key characteristic of sarcopenia with low muscle mass as a confirmatory factor. Low physical performance such as slow gait speed indicates severe sarcopenia. This update should improve the diagnosis of sarcopenia among hospitalised older patients similar to those in this study.

We found that it was feasible to assess frailty amongst older hospital patients using the Fried Frailty Phenotype (94%) and the FRAIL scale (99%). The Fried Frailty Phenotype can take up to 20 min to complete with a trained assessor while the FRAIL scale is a simple tool that takes up to 5 min to complete without the need for a trained assessor [44]. The Fried Frailty Phenotype has good construct validity [23], convergent validity [45], concurrent validity [46] and predictive validity [47] for assessing frailty. It has also been shown that the tool has good sensitivity to change following an intervention in frail patients [48]. There is evidence that FRAIL scale has an acceptable validity and reliability [27, 49, 50] and that it has similar predictive accuracy to the Fried Frailty Phenotype, in keeping with the moderate agreement reported in this study.

In our cohort of acutely unwell older people, the prevalence of frailty was 48% assessed by the Fried Frailty Phenotype, and 34% by the FRAIL scale, consistent with other studies of hospital patients such as a study of 495 patients in Canada which reported 43% to be frail [51]. The difference between the scales may reflect the objective measurements in the Fried and the subjective opinion of participants in the FRAIL scale. Sarcopenia according to the EWGSOP criteria was identified among 33% of the participants able to complete the assessments. Other studies using the EWGSOP criteria and bioelectrical impedance have reported sarcopenia among 41% male and 32% female Portuguese hospital patients aged > 65 years [41], 21.4% of Italian acutely unwell patients aged > 65 years [34] and 25% of German acute medical inpatients (mean age 83) [52]. However, the challenges of measuring muscle mass among hospitalised older people make it difficult to draw conclusive estimates of the prevalence of sarcopenia among this population.

### Strengths and limitations

A small team of trained researchers conducted all the assessments using standardised protocols and calibrated instruments whose accuracy was checked. The participants were well described and typical of a geriatric inpatient population with a median of 5 comorbidities, mostly living in their own homes. However, there were limitations to the study. The majority of participants were discharged home after a median stay of 10 days with a low mortality rate suggesting that this was a less ill group, which may reflect the need for informed consent to enter the study. Therefore, the prevalence of frailty and sarcopenia of this in-patient group may have been under-estimated. We considered inability to perform grip strength and gait speed as indicative of poor function as suggested in the literature, which may also have impacted the prevalence values reported here. This sample of participants from one hospital also had little ethnic diversity. We specifically evaluated the Fried Frailty Phenotype, FRAIL Scale and EWGSOP tools but additional comparison of other tools such as the Clinical Frailty Scale and SARC-F tool for screening sarcopenia would be helpful for future studies, which should investigate the feasibility of clinical staff completing the assessments to improve the generalisability of the results.

## Conclusion

This study examined the feasibility of assessing frailty and sarcopenia in hospitalised older people using the measurements required to underpin the widely validated Fried Frailty Phenotype and EWGSOP criteria. Measuring grip strength was feasible and almost all participants completed this assessment. Measuring gait speed and muscle mass among older acutely unwell patients was difficult and many were unable to complete these assessments. Imputing the inability to complete gait speed and grip strength assessments as low values, we were able to report the prevalence of frailty using the Fried Frailty Phenotype. The FRAIL scale was completed by 99% participants with moderate agreement between the two frailty measures. The difficulty of assessing muscle mass using bioelectrical impedance, made the identification of sarcopenia in this acute setting challenging. The results of this study support the updated EWGSOP2 guidelines with a focus on muscle strength as the key characteristic of sarcopenia.

## Abbreviations

ADL: Activities of Daily Living
BMI: Body Mass Index
DXA: Dual-energy X-ray absorptiometry
EWGSOP: European Working Group on Sarcopenia in Older People
GDS: Geriatric Depression Scale
ICD-10: International Classification of Diseases
MMSE: Mini Mental State Examination
PASE: Physical Activity Scale for the Elderly

## References

[1] Clegg A, Young J, Iliffe S, Rikkert MO, Rockwood K (2013). Frailty in elderly people. Lancet.

[2] Syddall H, Roberts HC, Evandrou M, Cooper C, Bergman H, Aihie SA (2010). Prevalence and correlates of frailty among community-dwelling older men and women: findings from the Hertfordshire cohort study. Age Ageing.

[3] Anker SD, Morley JE, von Haehling S (2016). Welcome to the ICD-10 code for sarcopenia. J Cachexia Sarcopenia Muscle.

[4] Theou O, Squires E, Mallery K, Lee JS, Fay S, Goldstein J (2018). What do we know about frailty in the acute care setting? A scoping review. BMC Geriatr.

[5] Lin HS, Watts JN, Peel NM, Hubbard RE (2016). Frailty and post-operative outcomes in older surgical patients: a systematic review. BMC Geriatr.

[6] Fougère B, Morley JE. Rapid Screening for Frailty and Sarcopenia in Daily Clinical Practice. J Nutr Health Aging. 2018;22:1023. 10.1007/s12603-018-1057-x.

[7] Bouillon K, Kivimaki M, Hamer M, Sabia S, Fransson EI, Singh-Manoux A (2013). Measures of frailty in population-based studies: an overview. BMC Geriatr.

[8] Bruyere O, Buckinx F, Beaudart C, Reginster JY, Bauer J, Cederholm T (2017). How clinical practitioners assess frailty in their daily practice: an international survey. Aging Clin Exp Res.

[9] Woo J, Yu R, Wong M, Yeung F, Wong M, Lum C (2015). Frailty screening in the community using the FRAIL scale. J Am Med Dir Assoc.

[10] Abellan van Kan G, Rolland Y, Bergman H, Morley JE, Kritchevsky SB, Vellas B (2008). The I.A.N.A Task Force on frailty assessment of older people in clinical practice. J Nutr Health Aging.

[11] Woo J, Leung J, Morley JE (2012). Comparison of frailty indicators based on clinical phenotype and the multiple deficit approach in predicting mortality and physical limitation. J Am Geriatr Soc.

[12] Cruz-Jentoft AJ, Baeyens JP, Bauer JM, Boirie Y, Cederholm T, Landi F. Sarcopenia: European consensus on definition and diagnosis: report of the European working group on sarcopenia in older people. Age Ageing. 2010;39(4):412–23. 10.1093/ageing/afq034. 10.1093/ageing/afq034 PMC2886201 20392703

[13] Locquet M, Beaudart C, Reginster J-Y, Petermans J, Bruyère O (2018). Comparison of the performance of five screening methods for sarcopenia. Clin Epidemiol.

[14] Yu SCY, Khow KSF, Jadczak AD, Visvanathan R (2016). Clinical Screening Tools for Sarcopenia and Its Management. Curr Gerontol Geriatr Res.

[15] Beaudart C, McCloskey E, Bruyère O, Cesari M, Rolland Y, Rizzoli R (2016). Sarcopenia in daily practice: assessment and management. BMC Geriatr.

[16] Ibrahim K, May CR, Patel HP, Baxter M, Sayer AA, Roberts HC (2018). Implementation of grip strength measurement in medicine for older people wards as part of routine admission assessment: identifying facilitators and barriers using a theory-led intervention. BMC Geriatr.

[17] Jadczak A, Mahajan N, Visvanathan R (2017). The feasibility of standardised geriatric assessment tools and physical exercises in frail older adults. J Frailty Aging.

[18] Howson FFA, Robinson SM, Lin SX, Orlando R, Cooper C, Sayer AAP (2018). Can trained volunteers improve the mealtime care of older hospital patients? An implementation study in one English hospital. BMJ Open.

[19] Folstein MF, Robins LN, Helzer JE (1983). The mini-mental state examination. Arch Gen Psychiatry.

[20] Sheikh JI, Yesavage JA (1986). Geriatric depression scale (GDS): recent evidence and development of a shorter version.

[21] Washburn RA, Smith KW, Jette AM, Janney CA (1993). The physical activity scale for the elderly (PASE): development and evaluation. J Clin Epidemiol.

[22] Fricke J, Unsworth CA (1997). Inter-rater reliability of the original and modified Barthel index, and a comparison with the functional Independence measure. Aust Occup Ther J.

[23] Fried LP, Tangen CM, Walston J, Newman AB, Hirsch C, Gottdiener J (2001). Frailty in older adults: evidence for a phenotype. J Gerontol A Biol Sci Med Sci.

[24] Roberts HC, Denison HJ, Martin HJ, Patel HP, Syddall H, Cooper C (2011). A review of the measurement of grip strength in clinical and epidemiological studies: towards a standardised approach. Age Ageing.

[25] Pritchard JM, Kennedy CC, Karampatos S, Ioannidis G, Misiaszek B, Marr S (2017). Measuring frailty in clinical practice: a comparison of physical frailty assessment methods in a geriatric out-patient clinic. BMC Geriatr.

[26] Cesari M, Kritchevsky SB, Newman AB, Simonsick EM, Harris TB, Penninx BW (2009). Added value of physical performance measures in predicting adverse health-related events: results from the health, aging and body composition study. J Am Geriatr Soc.

[27] Morley JE, Malmstrom TK, Miller DK (2012). A simple frailty questionnaire (FRAIL) predicts outcomes in middle aged African Americans. J Nutr Health Aging.

[28] Dodds RM, Granic A, Davies K, Kirkwood TB, Jagger C, Sayer AA. Prevalence and incidence of sarcopenia in the very old: findings from the Newcastle 85+ Study. J Cachexia Sarcopenia Muscle. 2016;8(2):229–37. 10.1002/jcsm.12157. 10.1002/jcsm.12157 PMC5377385 27897431

[29] Bodilsen AC, Juul-Larsen HG, Petersen J, Beyer N, Andersen O, Bandholm T (2015). Feasibility and inter-rater reliability of physical performance measures in acutely admitted older medical patients. PLoS One.

[30] Gariballa S, Alessa A (2017). Impact of poor muscle strength on clinical and service outcomes of older people during both acute illness and after recovery. BMC Geriatr.

[31] Graham JE, Ostir GV, Fisher SR, Ottenbacher KJ (2008). Assessing walking speed in clinical research: a systematic review. J Eval Clin Pract.

[32] Tucker PW, Evans DD, Clevenger CK, Ardisson M, Hwang U (2016). Feasibility of nurses measuring gait speed in older community-dwelling Emergency Department patients. Geriatr Nurs.

[33] Ostir GV, Berges I, Kuo YF, Goodwin JS, Ottenbacher KJ, Guralnik JM (2012). Assessing gait speed in acutely ill older patients admitted to an acute care for elders hospital unit. Arch Intern Med.

[34] Cerri AP, Bellelli G, Mazzone A, Pittella F, Landi F, Zambon A (2015). Sarcopenia and malnutrition in acutely ill hospitalized elderly: Prevalence and outcomes. Clin Nutr.

[35] Bruyère O, Beaudart C, Reginster J-Y, Buckinx F, Schoene D, Hirani V (2016). Assessment of muscle mass, muscle strength and physical performance in clinical practice: an international survey. Eur Geriatr Med.

[36] Dodds R, Sayer AA (2016). Sarcopenia and frailty: new challenges for clinical practice. Clin Med.

[37] Litwic A (2014). Practicalities of DXA in older adults: observations from the CASIO study. Osteoporos Int.

[38] Pagotto V, Silveira EA (2014). Methods, diagnostic criteria, cutoff points, and preva-lence of sarcopenia among older people. Sci World J.

[39] Yilmaz O, Bahat G (2017). Suggestions for assessment of muscle mass in primary care setting. Aging Male.

[40] Buckinx F, Landi F, Cesari M, Fielding RA, Visser M, Engelke K (2018). Pitfalls in the measurement of muscle mass: a need for a reference standard. J Cachexia Sarcopenia Muscle.

[41] Sousa AS, Guerra RS, Fonseca I, Pichel F, Amaral TF (2015). Sarcopenia among hospitalized patients - A cross-sectional study. Clin Nutr.

[42] Van Ancum JM, Pijnappels M, Jonkman NH, Scheerman K, Verlaan S, Meskers CGM (2018). Muscle mass and muscle strength are associated with pre- and post-hospitalization falls in older male inpatients: a longitudinal cohort study. BMC Geriatr.

[43] Cruz-Jentoft AJ, Bahat G, Bauer J, Boirie Y, Bruyère O, Cederholm T, et al. Writing Group for the European Working Group on Sarcopenia in Older People 2 (EWGSOP2), and the Extended Group for EWGSOP2. Sarcopenia: revised European consensus on definition and diagnosis. Age Ageing. 2019;48(1):16–31. 10.1093/ageing/afy169. 10.1093/ageing/afy169 PMC6322506 30312372

[44] Dent E, Kowal P, Hoogendijk EO (2016). Frailty measurement in research and clinical practice: a review. Eur J Intern Med.

[45] Avila-Funes JA, Medina-Campos RH, Tamez-Rivera O, Navarrete-Reyes AP, Amieva H, Aguilar-Navarro S (2014). Frailty is associated with disability and recent hospitalization in community-dwelling elderly: the Coyoacan cohort. J Frailty Aging.

[46] Rockwood K, Andrew M, Mitnitski A (2007). A comparison of two approaches to measuring frailty in elderly people. J Gerontol A Biol Sci Med Sci.

[47] Chang SF, Lin PL (2015). Frail phenotype and mortality prediction: a systematic review and meta-analysis of prospective cohort studies. Int J Nurs Stud.

[48] Li CM, Chen CY, Li CY, Wang WD, Wu SC. The effectiveness of a comprehensive geriatric assessment intervention program for frailty in community-dwelling older people: a randomized, controlled trial. Arch Gerontol Geriatr. 2010;50(Suppl 1):S39–42. 10.1016/S0167-4943(10)70011-X 20171455

[49] Dong L, Qiao X, Tian X, Liu N, Jin Y, Si H (2018). Cross-cultural adaptation and validation of the FRAIL scale in Chinese community-dwelling older adults. J Am Med Dir Assoc.

[50] Gardiner PA, Mishra GD, Dobson AJ (2015). Validity and responsiveness of the FRAIL scale in a longitudinal cohort study of older Australian women. J Am Med Dir Assoc.

[51] Belga S, Majumdar SR, Kahlon S, Pederson J, Lau D, Padwal RS (2016). Comparing three different measures of frailty in medical inpatients: multicenter prospective cohort study examining 30-day risk of readmission or death. J Hosp Med.

[52] Smoliner C, Sieber CC, Wirth R (2014). Prevalence of sarcopenia in geriatric hospitalized patients. J Am Med Dir Assoc.

